# The Implications of Membranes Used as Separators in Microbial Fuel Cells

**DOI:** 10.3390/membranes11100738

**Published:** 2021-09-28

**Authors:** Jonathan Ramirez-Nava, Mariana Martínez-Castrejón, Rocío Lley García-Mesino, Jazmin Alaide López-Díaz, Oscar Talavera-Mendoza, Alicia Sarmiento-Villagrana, Fernando Rojano, Giovanni Hernández-Flores

**Affiliations:** 1 Facultad de Ecología Marina, Universidad Autónoma de Guerrero, Gran vía Tropical No 20, Fracc. Las Playas, Acapulco 39390, Mexico; jonathanramirez@uagro.mx (J.R.-N.); 10110848@uagro.mx (R.L.G.-M.); 14558@uagro.mx (J.A.L.-D.); 2 Centro de Ciencias de Desarrollo Regional, Universidad Autónoma de Guerrero, Privada de Laurel No. 13, Col. El Roble, Acapulco 39640, Mexico; marianamartinez@uagro.mx; 3 Escuela Superior de Ciencias de la Tierra, Universidad Autónoma de Guerrero, Ex Hacienda San Juan Bautista s/n, Taxco el Viejo 40323, Mexico; otalavera@uagro.mx; 4 Facultad de Ciencias Agropecuarias y Ambientales, Universidad Autónoma de Guerrero, Periférico Poniente s/n, Frente a la Colonia Villa de Guadalupe, Iguala de la Independencia 40040, Mexico; 19247@uagro.mx; 5 Gus R. Douglass Institute, West Virginia State University, Institute, WV 25112, USA; fernando.rojano@wvstateu.edu; 6 CONACYT-Escuela Superior de Ciencias de la Tierra, Universidad Autónoma de Guerrero, Ex Hacienda San Juan Bautista s/n, Taxco el Viejo 40323, Mexico

**Keywords:** membranes, microbial fuel cell, proton-exchange membrane, separators

## Abstract

Microbial fuel cells (MFCs) are electrochemical devices focused on bioenergy generation and organic matter removal carried out by microorganisms under anoxic environments. In these types of systems, the anodic oxidation reaction is catalyzed by anaerobic microorganisms, while the cathodic reduction reaction can be carried out biotically or abiotically. Membranes as separators in MFCs are the primary requirements for optimal electrochemical and microbiological performance. MFC configuration and operation are similar to those of proton-exchange membrane fuel cells (PEMFCs)—both having at least one anode and one cathode split by a membrane or separator. The Nafion^®^ 117 (NF-117) membrane, made from perfluorosulfonic acid, is a membrane used as a separator in PEMFCs. By analogy of the operation between electrochemical systems and MFCs, NF-117 membranes have been widely used as separators in MFCs. The main disadvantage of this type of membrane is its high cost; membranes in MFCs can represent up to 60% of the MFC’s total cost. This is one of the challenges in scaling up MFCs: finding alternative membranes or separators with low cost and good electrochemical characteristics. The aim of this work is to critically review state-of-the-art membranes and separators used in MFCs. The scope of this review includes: (i) membrane functions in MFCs, (ii) most-used membranes, (iii) membrane cost and efficiency, and (iv) membrane-less MFCs. Currently, there are at least 20 different membranes or separators proposed and evaluated for MFCs, from basic salt bridges to advanced synthetic polymer-based membranes, including ceramic and unconventional separator materials. Studies focusing on either low cost or the use of natural polymers for proton-exchange membranes (PEM) are still scarce. Alternatively, in some works, MFCs have been operated without membranes; however, significant decrements in Coulombic efficiency were found. As the type of membrane affects the performance and total cost of MFCs, it is recommended that research efforts are increased in order to develop new, more economic membranes that exhibit favorable properties and allow for satisfactory cell performance at the same time. The current state of the art of membranes for MFCs addressed in this review will undoubtedly serve as a key insight for future research related to this topic.

## 1. Introduction

Nowadays, fossil fuels are extensively; however, they are not a renewable source of energy. Furthermore, fossil fuels have caused social and environmental problems [1,2]. To mitigate these effects, research efforts supporting renewable energy sources can suggest alternatives [3,4,5], notwithstanding the limitations of natural resources in certain regions of the globe. The feasibility of improving the performance of these renewable energy sources relies on integrating suitable and efficient energy storage systems (zero CO_2_ emissions) capable of storing unstable energy generated through options such as lithium-ion batteries [6], iron-based redox flow batteries [7], and super-capacitors [8]. Integration of these systems requires trade-offs between the physical and chemical fundamentals of energy generation and its storage through advanced systems beyond the capacity of fuel cells (FCs).

At the beginning of the 19th century, FCs appeared as a green energy technology. FCs represent a way of producing clean energy, which implies zero CO_2_ emissions. These electrochemical systems transform the chemical energy stored in H_2_ into electricity (Equation (1)), with water (Equation (2)) and large amounts of heat as by-products (Equation (3)), formed as a result of the oxidation–reduction process [9].

(1) $$H_{2}\to 2H^{+}+2e^{-}\;\;\;\mathrm{Hydrogen}\;\mathrm{oxidation}$$

(2) 12O2+2H++2e−→H2O   Oxygen reduction

(3) H2+12O2→H2O+ΔH°f=−285.8 kJ/mol   Global oxidation–reduction reaction

This type of electrochemical system is a kind of emerging technology whose application is not limited by geographical restrictions [10]. A century following its invention, the concept of this new technology explored the development of microbial fuel cells (MFCs), a particular type of bioelectrochemical system also considered to be a green eco-friendly technology [5,11,12]. Background research into this novel concept was introduced by Potter [12]; he observed an electrical current generated by microorganisms in the presence of organic compounds [13,14]. The concept of MFCs suggests the existence of a renewable alternative energy source. Recently, in the last two decades, research interest in MFCs began to grow [15,16,17,18,19,20,21,22,23]. In this type of system, pure organic compounds or complex mixtures of dissolved organic matter in wastewater or leachates are used as fuel. The chemical energy of the compounds is transformed into electricity through an oxidation–reduction process [19,21,24]. Yielding of the energy from oxidation of the substrates is carried out by microorganisms under anoxic conditions, which are commonly referred to as biocatalysts [18,25,26]. From this overview, MFCs are considered an interesting proposal for both electrical energy recovery and wastewater treatment, at the same time [14]. However, an MFC comprises a set of engineering variables that require evaluation [18]; a wide variety of scientific and engineering disciplines are needed to succeed in the design and operation of MFCs [14,27]. As commonly happen in other technologies, researchers are working towards scaling these devices and improving performance by producing higher energy power and reducing costs [28,29]. Despite these research efforts, some factors still limit MFCs’ practical application [25,30]; some include the electrode material and surface area, the catalyst used at the cathode and the biocatalysts at the anode, the total internal resistance of the MFC ($R_{int}$) and the external resistance used, along with the operation, the electric conductivity of the anolyte and catholyte, the distance between the anode and cathode, and the membrane type, among others [18,28,31,32,33]. However, the membrane type is one of the most important factors in the development of MFCs, and it represents around 60% of the MFC’s total cost [23,25,33,34,35]. The presence or absence of these components is directly reflected in the MFC performance, e.g., power density (*P*), and cost.

Nowadays, Nafion^®^ 117 (NF-117) membranes have the optimal characteristics required for MFCs [18,25]. However, despite the fact that NF-117 membranes the best available membranes for MFCs, their high price discourages their use once MFCs are scaled-up, limited by increases in $R_{int}$ [36,37]. At the time of choosing a membrane for MFC applications, it should meet several selection criteria such as outstanding mechanical and chemical stability, no electronic conduction, impermeability to gases such as $H_{2}$ and $N_{2}$, partial hydrophilicity, easy acquisition, high ionic conductivity, high species selectivity, low oxygen and fuel crossover, and low cost and electrical resistance [10,38,39,40]. Undesirably, membrane use increases the $R_{int}$ of the MFC due to the influence of the thickness of the membrane. Phenomena such as biofouling and fuel crossover also contribute to increases in the $R_{int}$ of MFCs during their operation. Sun and Zhang [41] demonstrated the influence of membrane thickness on some physicochemical properties; they used three commercial Nafion^®^ membranes: Nafion^®^ 212, Nafion^®^ 115, and NF-117, with different thicknesses 50, 126, and 178 μm, respectively. They observed that as the membrane thickness increases, the membrane resistance increases, and the proton conductivity decreases; however, a greater thickness is useful for restraining interpenetration of electro-active species. Additionally, they observed that the three membranes evaluated with different thicknesses showed similarly high levels of chemical stability. Therefore, high cost and the influence of membrane thickness on the $R_{int}$ of MFCs are two big challenges to overcome. In search of new membranes or separators able to provide/produce a similar performance to Nafion^®^ and reduce cost, many alternatives have been studied. Some of the membranes and separators assessed in MFCs by researchers are the following: cation-exchange membranes (e.g., sulphonated poly(ether ether ketone membranes *SPEEKs*)), Selemion HSFs, and polystyrene and divinylbenzene with sulfonic acid groups), anion-exchange membranes (e.g., Zirfon^®^), ultrafiltration and microfiltration membranes, bipolar membranes, forward osmosis membranes, cloth (J-cloth) separators, glass fiber separators, cation-exchange layers made of purified kaolin, porous porcelain coated with Nafion^®^ 117 solution, dialysis membranes, thin layer spray-coating of hydrophilic cation-exchange polymers, anion-exchange and neutral polymers, porous fabrics and coarse-pore filter material, polytetrafluoroethylene membranes, isopore membrane filters, biomax ultrafiltration discs, glass wool, nylon membranes, polycarbonate membranes, cellulose nitrate membranes, kaolin, porcelain and polyethylene membrane interpolymers, forward osmosis membranes, and agar–agar membranes [1,2,16,18,25,27,31,37,38,42,43,44,45,46,47,48,49,50]. The aim of these studies is to lessen costs, reduce $R_{int}$, increase *P* output and Coulombic efficiency ($\eta_{coul}$), and to improve the membrane separator as a key component [50]. 

This work aims to critically review the state of the art on membranes and separators used in MFCs and their implications. The scope of this work includes the review of (i) membrane and separator functions in MFCs, (ii) the most-used membranes, (iii) membrane cost and efficiency, and (iv) membrane-less MFCs. 

## 2. Microbial Fuel Cell Components and Basic Functioning

**Figure 1:**
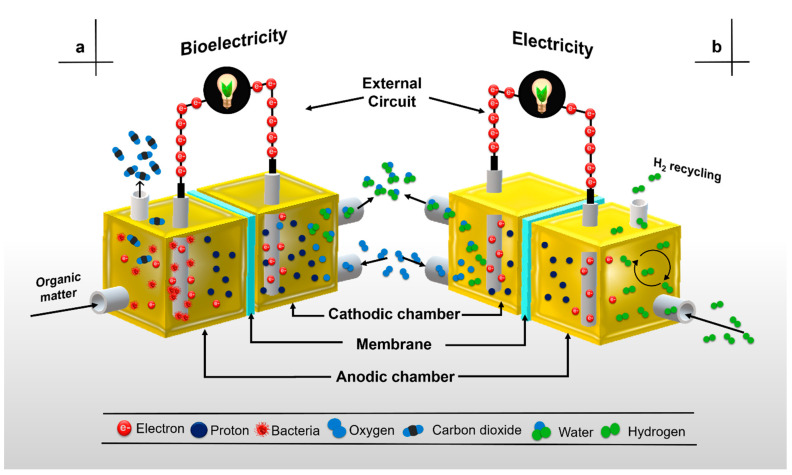
Basic components in (**a**) a microbial fuel cell and, (**b**) a fuel cell.

The basic principle of any electrochemical system is a physicochemical oxidation process coupled with a reduction process. An oxidation–reduction reaction is a physicochemical process involving a flow of electrons where an element or compound gets oxidized by another element/compound that gets reduced [9]. However, unlike oxidation–reduction reactions that naturally and spontaneously develop within the same system, the configuration of electrochemical systems requires oxidation and reduction reactions to be separated by a “membrane or separator”. The main objective of this membrane is to prevent oxidation and reduction reactions happening in the same place, i.e., if the redox reaction occurs in the same place, it will get short-circuited. On the other hand, the membrane must have the ability to function as a channel, allowing the flow of ionic species generated by oxidation, while generated electrons migrate through an external circuit (e.g., a platinum wire) from the anode to the cathode. This setup for performing the oxidation–reduction process defines the electrochemical system, generating a potential difference (Δ*V*) that can be converted into electrical energy [51]. The operation of MFCs depends on the separation of the two reactions, i.e., the gap between electrodes is a requirement, and the use of a membrane or separator is essential for the configuration. Additionally, at least one pair of electrodes, an anode and a cathode, joined via an external circuit, form part of the basic elements required in MFCs [11,23]. However, the biological activity of microorganisms/enzymes is also one of the basic elements constituting MFCs (Figure 1).

Bacteria, a minimum of one anode and one cathode, organic compounds used as fuel, an external circuit, and one membrane or separator are the elements commonly found in the two main types of MFCs configurations: single-chamber MFCs (SC-MFCs) and dual-chamber MFCs (DC-MFCs). In an SC-MFC, the anode electrode is inserted into the anodic chamber, interacting with microorganisms and their oxidative metabolism, whereas the cathode is exposed to air flow by natural convection. On the other hand, in a DC-MFC the anodic and cathodic electrodes are inserted into their respective chambers—one anodic and one cathodic [52,53]. Regardless of the configuration, MFC performance results show that the anodic and cathodic sections must be separated. NF-117 membranes separating the two chambers are one of the most commonly used proton exchange membranes (PEMs) available, and the high proton conductivity (PC) values exhibited by them makes them the choice of researchers for use in fuel cells and MFCs [5,10,18,27,49,54].

**Figure 2:**
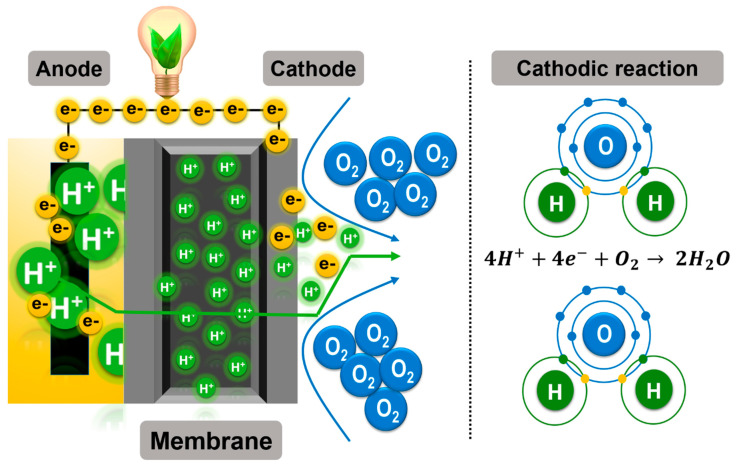
Oxygen reduction reaction at the cathode.

In the anodic compartment, the biocatalysts anaerobically oxidize the substrate and release electrons and protons. The electrons are collected by the anode and travel to the cathode via an external circuit [11]. Protons diffuse through the alcohol and MFC PEM to reach the cathodic section. At the cathode, the protons react with the electrons and produces water from the molecular oxygen in air. This reaction is known as the oxygen reduction reaction (ORR). This reaction is explained graphically in Figure 2 [1,2]. 

### 2.1. Electrochemical Membrane Concept

In general, a membrane acts as a thin physical barrier with a <200 µm thickness; it separates the fluids between the anodic and cathodic chambers, defined as anolyte and catholyte, respectively, where oxidation and reduction reactions take place. Nevertheless, complete separation is not observed. 

The membrane should not allow a physical interaction between the respective electrolytes, but rather should only aid in the transfer of ions (anions and/or cations) via electro-osmotic drag between the two MFC sections. Membranes must inhibit mass transfer between chambers; membrane performance depends on their physical and chemical properties. In the case of membranes with pores in their structure, membrane performance is the function of pore size and the number of pores (porosity). Nevertheless, there are nonporous membranes where porosity is conceptualized as the phase-separation degree between hydrophobic and hydrophilic phases, playing a significant role in membrane performance. The size of ion clusters (size of ion transport channel/pathways), and ion-exchange capacity (IEC) are other important factors to consider when evaluating nonporous membrane performance. Porous membranes do not have functional groups; therefore, they do not have IEC [55]. Thus, depending on the presence of pores, membranes have been classified into two groups: porous and nonporous membranes. The nonporous membranes, also called ion-exchange membranes (IEMs), are in turn classified into three groups based on the type of ion that is transferred: cation-exchange membranes (CEMs), anion-exchange membranes (AEMs), and bipolar membranes (BPMs). On the other hand, porous membranes have been grouped into ultrafiltration membranes (UFMs), microfiltration filtration membranes (MFMs), ceramic membranes (CMs), and pore filter materials [10,25,39].

### 2.2. Membrane Separator Functions in Microbial Fuel Cells

**Figure 3:**
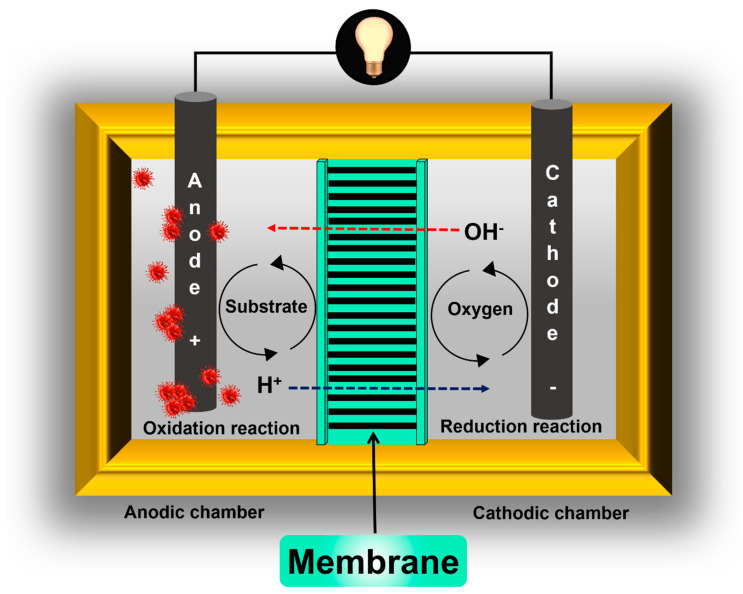
Membrane functions.

A membrane separator has several important functions in MFCs. As was previously mentioned, its purpose is to prevent short-circuiting between the electrodes and to separate their corresponding chemical reactions (Figure 3). On the other hand, the membrane must act as a channel conducting ions (protons, anions, or both) from the anode to the cathode or vice versa, and must inhibit organic compound (used as fuel) crossover from the anode to the cathode as well as electron acceptor crossover, such as oxygen, from the cathode to the anode, i.e., the membrane must have a high species selectivity. A high ionic conductivity could be considered as the second most important role of the membrane in MFCs. In the case of IEMs, they should have partially hydrophilic properties in order to provide ion conduction channels. However, for porous membranes, hydrophilic properties are not necessary, i.e., they can be fully hydrophobic.

In the case of SC-MFCs or DC-MFCs where oxygen is used as the final electron acceptor for the cathodic reaction, the membrane must inhibit oxygen diffusion (OD) from the cathode to the anode. This function of the membrane (high species selectivity) contributes to maintaining an anaerobic environment in the anodic section, necessary for sustaining an acceptable $\eta_{coul}$ and for the survival of electrochemically active bacteria [39,56]. In the case of PEMs, it must also avoid the transfer of other electron acceptors such as sulfate, ammonia, ferricyanide, permanganate, hydrogen peroxide, nitrate, trichloroethene, perchlorate, and some heavy metals that can alter the anodic microbial community, favoring the proliferation of non-electrochemically active microorganisms. The latter would lead to a reduction in $\eta_{coul}$ [18,57,58,59].

A similar phenomenon related to the substrate used as fuel in the anode chamber should be avoided: “fuel crossover”. In this case, the function of the membrane is to prevent soluble low molecular weight organic compounds from being transferred from the anode to the cathode (Figure 3) [18]. Additionally, another function of the membranes used in MFCs that is poorly discussed is in avoiding the crossing or exchange of microorganisms between chambers. In general, the success of a membrane within an MFC will depend mainly on its transport characteristics, i.e., it will depend on its ability to inhibit the transfer of certain species between chambers (substrate and oxygen) but allow the passage of others (protons or anions). 

On the other hand, the fuel usually used as the anolyte in MFCs has several organic and sometimes inorganic species. Additionally, microorganisms are essential fuel components—they are the catalyzers that carry out the oxidation process that occurs at the anodic chamber. Therefore, the membrane should have good chemical stability to prevent membrane oxidation and microbiological degradation [53]. 

## 3. Disadvantages of Using Membranes in Microbial Fuel Cells

Ideally, every membrane used in an MFC should meet all the characteristics described in Section 2.2. However, depending on its chemical composition and the physicochemical and microbiological characteristics of the anolyte and catholyte, membranes present certain function limitations. Some of the disadvantages related to MFC performance associated with the use of membranes are the following: MFC $R_{int}$ increase, OD from the cathode to the anode chamber, substrate crossover from the anode to the cathode chamber, biofouling, pH splitting, water loss by evaporation, and undesirable ion crossing [10,40].

### 3.1. Increase in the Total Internal Resistance of the Microbial Fuel Cell

The total $R_{int}$ of an MFC is one of the main factors related to the generation of current (*I*) and *P*. The total $R_{int}$ of an MFC is the sum of the resistances caused by design factors and physico-chemical properties of the materials used in the construction of the MFC, e.g., the materials for the electrodes and the separation between them, conductivity of the electrolyte, and factors related to the membrane [10,40].

The membrane itself, depending on its nature (organic, inorganic, or mixed compound) and the number of pores, contributes significantly to increasing the total $R_{int}$ of an MFC. The value of the membrane resistance (*R*) is associated with the IEC. The IEC is altered by system operating conditions: temperature, electrolyte type, pH, and concentration of the electrolyte solution. A low proton diffusion from the anode to the cathode will be reflected by low MFC performance. The value of *R* in an FC and an MFC is mainly attributed to the ohmic resistance (OR). There are several techniques to determine the OR value in electrochemical systems; however, the most common are the current interruption (CI) and electrochemical impedance spectroscopy techniques. The CI technique is also used to determine the value of *R*. The fundamentals of this technique are the interruption of the current in the cell and measurement of Δ*V* before and after the interruption. Following this, the OR of the membrane is calculated using Ohm’s law (Equation (4)) [40]:

(4) $$R=\frac{\Delta V}{I}$$

where *R* represents the OR of the membrane (Ω), Δ*V* is the voltage difference (*V*), and *I* the current (*A*). A high value of *R* has a negative impact on the MFC’s performance. A high value of $R_{int}$ will be reflected in a loss of *I* and *P* production (Equations (5)–(7)). The values of these parameters are important because they are part of the group of values considered when measuring MFC performance [53].

(5) $$I_{MFC}=\frac{E_{MFC}}{R_{int}}$$

where $I_{MFC}$ is the current of the MFC (*A*), $E_{MFC}$ represents the voltage generated by the MFC (*V*), and $R_{int}$ represents the total internal resistance of the MFC.

(6) $$P_{MFC}=\frac{E_{MFC}^{2}}{R_{int}}$$

(7) $$P_{MFC}=E_{MFC}I_{MFC}$$

where $P_{MFC}$ represents the power generated by the microbial fuel cell (*W*).

Another parameter affected by the value of *R* is the ionic conductivity (σ). This value is inversely proportional to *R*, i.e., the higher the *R* the lower the σ (Equation (8)).

(8) $$\sigma =\frac{L}{RA}$$

where σ represents the membrane ionic conductivity (S/cm), *L* is the membrane thickness (cm), *R* is the membrane resistance (Ω), and *A* is the area of the electrode (cm^2^).

**Figure 4:**
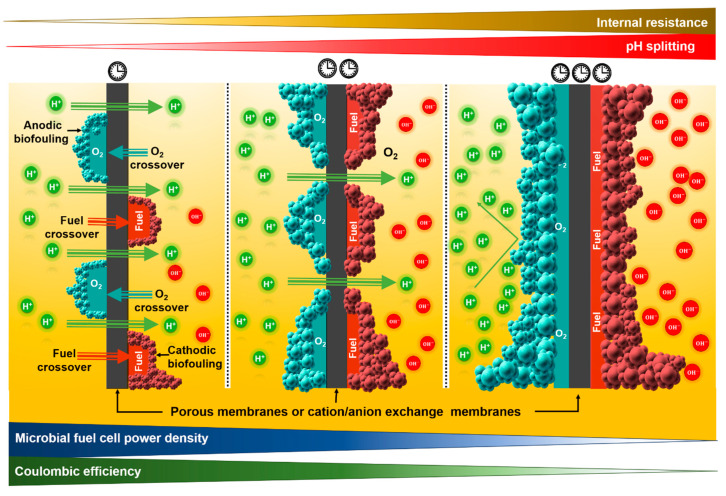
Membrane-use disadvantages in microbial fuel cells.

There are membranes with a low *R* value used as porous membranes, e.g., microfiltration membranes. However, a low *R* value does not reflect positive MFC performance results. This occurs because this type of membrane has high oxygen and fuel crossover values that are translated into low MFC performance in terms of $\eta_{coul}$ and *P* (Figure 4) [40]. On the other hand, nonporous membranes contribute negatively to MFC performance due to their high value of *R* shown. Despite this disadvantage, using this type of membrane instead of porous membranes with low *R* values is usually chosen. However, the use of thinner nonporous membranes to reduce the *R* value is not recommended, because, as previously discussed, as the thickness of a membrane decreases, the resistance decreases and the ion conductivity increases. Considering only these two parameters, it is possible to consider the use of thinner IEMs. Nevertheless, Sun and Zhang [41] demonstrated that thinner membranes such as Nafion^®^ 212 and Nafion^®^ 115, in comparison to NF-117, have a higher permeability to electro-active species—an undesirable effect. Moreover, usually, thinner IEMs have lower mechanical properties. Thus, the use of thinner nonporous membranes will not improve the overall membrane performance.

### 3.2. Oxygen Diffusion

The prevailing oxidizing agent in air is oxygen. It is the main final electron acceptor chosen to close the circuit at the cathode because of its high redox potential (+1.23 V vs. RHE) [9,53]. However, the presence of oxygen in the anodic chamber negatively affects the MFC’s performance [46]. First, its presence is toxic for anaerobic microorganisms that work as biocatalysts in the anode chamber. Furthermore, thermodynamically, it is the principal acceptor of electrons. In the case of facultative bacteria (also part of the group of biocatalysts present in the anode), dissolved oxygen will be the first choice of electron acceptor before the anodic substrate. The presence of oxygen at the anode strongly competes with the anodic material for the released electrons due to the anaerobic oxidation process that takes place. Consequently, $\eta_{coul}$ is an important parameter for measuring if MFC performance is affected as well [34,40,46].

One of the functions of the membrane is in preventing the diffusion of oxygen from the cathode chamber to the anode chamber. Unfortunately, nonporous membranes (including Nafion^®^ membranes) fail to meet this requirement. That is because oxygen has a significant solubility in water. For instance, Qu et al. [60] used an SC-MFC air cathode with a membrane filter as separator and a pure culture of *Geobacter sulphurreducens*, where it was found that the dissolved oxygen concentration at the anode reached ca. 6 mg/L, a concentration close to oxygen saturation levels. Consequently, growth of *G. sulfurreducens* was inhibited. The ion-exchange functionality in nonporous membranes is maximized when the membranes have been fully hydrated. Therefore, OD is associated with the need to keep the membrane hydrated. OD is the mechanism through which oxygen diffuses through the water and the membrane structure at the same time, towards the anode where the oxygen concentration is lower [39,40].

Among all these issues, the $\eta_{coul}$ is a parameter that must be considered for MFC performance and is defined as the transfer efficiency of available electrons to the anode; it is the total Coulombs transferred to the anode from the substrate, divided by the maximum Coulombs possible if all substrate removal were converted into *I* [1,15,18,25]. The $\eta_{coul}$ is calculated as follows (Equation (9)) [61]:

(9) $$\eta_{coul}\left(\%\right)=\frac{ACS}{TCS}\times 100$$

where *ACS* is the actual charge transferred from the substrate obtained by Equation (10), and the *TCS* is the maximum theoretical charge transferred from the substrate calculated by Equation (11).

(10) $$ACS=\int_{t=0}^{t=t}I_{MFC}dt$$

(11) $$TCS=\frac{F\times b_{COD}\times \left(COD_{i}-COD_{f}\right)\times V}{M_{COD}}$$

*I_MFC_* is the current over time delivered by the microbial fuel cell, *F* is Faraday’s constant: 96,485.33 Coulombs/mol *e^−^*, *b_COD_* is the number of electrons (4) exchanged per mole of oxygen generated by the chemical oxygen demand (*COD*), initial *COD* is *COD_i_* (g/L), final *COD* is *COD_f_* (g/L), *V* is the volume of liquid in the anode compartment (L), and *M_COD_* is the molecular weight of oxygen (32 g O_2_/mol *COD*).

However, the $\eta_{coul}$ is adversely affected by other factors such as bacterial growth, competitive processes, and the utilization of alternate electron acceptors by the microorganisms [1].

In general, porous membranes have a greater tendency to allow the passage of oxygen from the cathode to the anode due to the presence of pores compared to nonporous membranes (Figure 4). However, considering that IEMs need to be hydrated to acquire their functionality, it will be difficult to synthesize a membrane that avoids OD entirely.

### 3.3. Substrate Crossover

In theory, nonporous membranes do not allow nonionic species to cross to the cathode. However, in the anode, different pure compounds such as acetates, butyrates, and propionates, and wastewater with a large amount of dissolved low molecular weight organic compounds, have been evaluated as fuels or substrates resulting in susceptibility to the substrate/fuel crossover phenomenon. It is a similar phenomenon to that experienced by oxygen (oxygen diffusion), but in the opposite direction. This phenomenon is observed when dissolved organic molecules are used as a source of carbon and energy by biocatalysts, i.e., the organic compounds or organic matter present in wastewater diffuse through the membranes from the anaerobic to the aerobic cathode chamber (Figure 4) [10,39,40]. Once again, water is the solvent of the anolyte and, therefore, the hydrophilic nature of the membrane favors the embedding of this solvent into the membrane and crossover of dissolved organic molecules towards the cathode as a function of the current concentration gradient. Unlike ions, organic compounds are considerably larger. Therefore, in the case of nonporous membranes, the occurrence of this process (substrate loss) is practically nil, except in AEMs. AEMs are characterized by having positively charged ionic groups. When some simple acids that are used as substrates, e.g., acetate, propionate, or their mixtures, are deprotonated, they acquire a negative charge at pHs close to neutrality. This change in the charge of the substrate favors interactions with chemical charges present at the membrane. Consequently, negatively charged organic compounds diffuse across AEMs at a slower rate. On the other hand, in the case of porous membranes with large pore sizes, the substrate crossover phenomenon takes place at a higher speed through the pores compared to AEMs. 

When the substrate migrates from the anodic to the cathodic chamber, several effects are observed: (i) the amount of substrate available at the anode for the biocatalysts decreases, (ii) the substrate is oxidized at the cathode by aerobic bacteria producing electrons for the ORR that is carried out at the cathode, (iii) biofouling is generated at the cathode surface and reduces oxygen interactions with the cathode active surface, (iv) the *P* decreases, and (v) the $\eta_{coul}$ decreases as a consequence of each of these mentioned activities [10,39,40]. 

### 3.4. Biofouling

This phenomenon is generally observed in MFCs where oxygen is the oxidizing agent used at the cathode. It is characterized by the adherence of organic compounds used as sources of carbon and energy, and by microorganisms on the membrane surface, exposed towards the interior of the anode section (anodic biofouling). Anodic biofouling will appear mainly in MFCs that use nonporous membranes. It begins with the adhesion of organic compounds on the surface of the membrane facing the interior of the anode section; subsequently, OD through the membrane from the cathode to the anode, combined with long periods of operation of the MFCs, favors the proliferation of aerobic microorganisms on the membrane (Figure 4) [39,40]. 

Organic matter oxidation processes, under aerobic conditions, are carried out at a higher speed than anaerobic oxidation processes. Also, in this type of process, the largest possible amount of energy (ca. 65%) is applied to generate new cells that will be translated into a greater quantity of sludge, while in anaerobic conditions, the amount of energy employed to create new cells is considerably lower (ca. 10%) [62]. This aerobic and oxidative microenvironment generated on the surface of the membrane causes: (i) substrate consumption at a higher speed susceptible to conversion into electrical energy, (ii) a negative oxygen gradient due to the aerobic bacteria demand; the latter leads to more oxygen passing from the cathode to the anode, and (iii) an additional barrier between the anolyte and the membrane—a product of the biofilm generated by organic compounds and microorganisms—increasing $R_{int}$ and *R*, and decreasing σ (Equation (8)). This decrease in σ favors the acidification of the anolyte and causes a pH gradient between the chambers. The sum of all these effects will be reflected in an MFC performance decrease in terms of *P_MFC_* and $\eta_{coul}$, as shown in Equations (6) and (9) [40]. Biofouling can also be observed on the surface of the membrane exposed towards the cathode (cathodic biofouling). In this case, fuel crossover is the factor that will favor biofouling on the surface of the membrane exposed to the cathodic section (Figure 4). Here, aerobic microorganisms oxidize the organic compounds generating electrons for the ORR that are not provided through the external circuit, i.e., the oxidation–reduction process is carried out within the same system, generating an internal short circuit that will decrease the *P_MFC_* and $\eta_{coul}$. Depending on the porosity, cathodic biofouling will be mostly observed in porous membranes. However, in AEMs (nonporous membranes), this phenomenon has also been observed, although to a lesser extent, since some low molecular weight organic acids can cross the membrane [10].

Biofouling is a process that depends on fuel crossover, OD, membrane porosity, and operating time. The greater the fuel or oxygen crossover and operating time, the greater the thickness of the biofouling will be. This will increase the thickness of the membrane, and as a direct consequence *R* will increase; therefore, the MFC performance will decrease [10].

### 3.5. pH Splitting

This phenomenon associated with the use of electrode-separating membranes is characterized by a wide variation in pH between the anodic and cathodic chambers during MFC operation, i.e., a high pH gradient between chambers can be observed after an operation period. Depending on the type of membrane selected, and the characteristics of the anolyte, the observed pH splitting will be as shown in Figure 4. For instance, the combination of a cation-rich anolyte (10^5^ times higher than the $H^{+}$ concentration) such as $NH_{4}^{+}$, $Na^{+}$, $K^{+}$, $Mg^{2+}$, and $Ca^{2+}$, and a CEM, leads to splitting effects compared to the use of an AEM. The high concentration of cations will compete directly against the crossing of the $H^{+}$. These cations will first pass before the $H^{+}$, causing an accumulation in the anode and, therefore, medium acidification. Under acidic conditions, anaerobic bacterial oxidation is inhibited by decreasing the proton and electron generation. Besides, in the absence of $H^{+}$ transfer to the cathode, ORR is carried out at a considerably low rate. Consequently, the pH of the catholyte increases. This phenomenon considerably decreases MFC performance. AEMs represent a good option for eliminating this phenomenon; the proton transfer rate is not limited, because the anions of the AEM are responsible for the transfer of $H^{+}$. Therefore, there are no other cations that can compete with the $H^{+}$ that adhere to the surface of the AEM. In MFCs using this type of membrane, pH splitting is practically not observed [10,40].

### 3.6. Water Loss by Evaporation

The membrane’s chemical composition makes it partially hydrophilic, and this favors σ. However, this characteristic also facilitates water transport and water evaporation and becomes a design problem, especially in SC-MFCs. One study reports an estimate that for every $H^{+}$ transferred to the cathode, 3H_2_O molecules pass through the membrane [39]. Hernández-Flores et al. [63] observed a considerable loss of water volume when comparing two single-chamber devices in operation: the first one consisted of an SC-MFC using NF-117 as a PEM, while the second SC-MFC was operated using Zirfon^®^ as an AEM. In both designs, the cathode section aerated by natural convection and exposed to an environment with a low percentage of humidity favored the loss of water by evaporation.

### 3.7. Undesirable Ions Crossing

**Figure 5:**
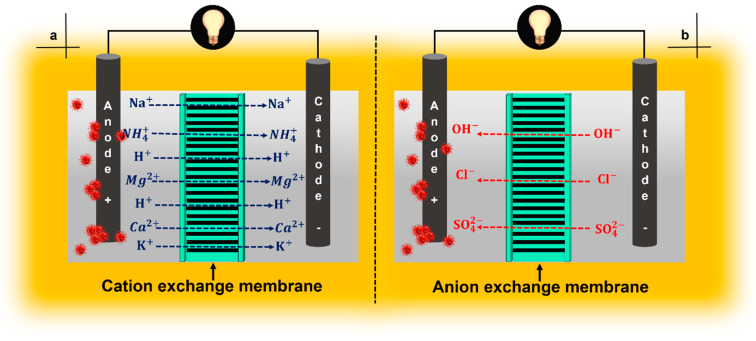
Main ions transported in a microbial fuel cell working with (**a**) a cation-exchange membrane and (**b**) an anion-exchange membrane.

In the case of nonporous membranes or IEMs specifically, in CEMs and AEMs, for $H^{+}$ and $OH^{-}$, respectively, the permeability to their corresponding ions is not 100% efficient; that depends on the concentration of other ions present in the anolyte and catholyte. In the case of CEM, the presence of other cations $NH_{4}^{+}$, $Na^{+}$, $K^{+}$, $Mg^{2+}$, and $Ca^{2+}$ will compete with $H^{+},$ and there is a possibility that they will cross the membrane and generate pH splitting (Figure 5a). Besides, in the case of AEM, anions other than $OH^{-}$, such as $Cl^{-}$ and $SO_{4}^{2-}$, can pass through these membrane types (Figure 5b). The transfer of ions other than those desired becomes a problem throughout the operation of the membrane system [10,38].

## 4. Ion-Exchange Membranes

### 4.1. Cation-Exchange Membranes

CEMs are characterized by their allowance of the passage of “positive ions” through them. Their chemical composition is characterized by the presence of negative charges (anions) such as $SO_{3}^{-}$, $COO^{-}$, $PO_{3}^{2-}$,$\;HPO_{3}^{-}$, and $C_{6}H_{4}O^{-}$, among others, when the membrane is hydrated [10]. Membranes such as Nafion^®^ 112, NF-117, Hyflon^®^, Zirfon^®^, Ultrex, and CMI-7000 are some types of membrane that have been used in MFCs as CEMs [10,40,64,65]. Although Nafion^®^ 212 and 115 have better PCs (0.092 and 0.088 S/cm, respectively, at 25 °C) than NF-117 (0.086 S/cm), NF-117 is the membrane most widely used in MFCs, probably because this membrane (thicker than Nafion^®^ 212 and 115) possesses relatively lower permeability to electro-active species. The thicknesses reported for these membranes are 55, 181, and 211 μm (wet conditions) for Nafion^®^ 212, Nafion^®^ 115, and NF-117, respectively [41]. 

The negatively charged sulfonate functional group is attached to the hydrophobic structure of fluorocarbon; the hydrophilic nature of the sulfonate group promotes the transport of protons through the membrane [10,40]. In addition to its high PC, it has a low OR that translates into a lower MFC $R_{int}$ and high *P* values. Ultrex CMI-7000 membranes have also been found to produce power densities similar to those of NF-117 [40]. These types of membranes are alternative candidates for use in MFCs. Despite the excellent characteristics and results that have been obtained with the use of CEMs, several problems have been observed during their use. Among them, the difference in pH between the anodic and cathodic chambers as a consequence of $H^{+}$ accumulation at the anode (pH splitting), diffusion of oxygen from the cathode to the anode, loss of substrate, and biofouling cause a decrease in σ [40,65,66]. Ideally, the membrane should be permeable only to $H^{+}$, however other cation species such as $NH_{4}^{+}$, $Na^{+}$, $K^{+}$, $Mg^{2+}$, and $Ca^{2+}$ compete against the passage of $H^{+}$, generating an increase in acidity at the anode (Figure 5a). This effect has been reported in previous studies [10,38].

#### Nafion^®^ 117 Membrane Properties

NF-117 belongs to the group of CEMs. The purpose of this membrane is to be selectively permeable to $H^{+}$. From this characteristic, NF-117 can also be identified as a PEM. The properties of NF-117 have positioned it as one of the most-used membranes in FCs and MFCs, due to its high σ PC value > 9.5 mS/cm (Table 1). NF-117 belongs to a group of membranes made from the polymer called perfluorinated sulfonic acid (PFSA), based on a polytetrafluoroethylene (PTFE) backbone with perfluorinated-vinyl-polyether side chains [67].

DuPont Corporation was the first to produce PTFE in 1938, with the trademark Teflon^TM^ [68]. However, PTFE is a strongly hydrophobic material, so to make an ion-conducting membrane, it must be chemically treated a second time. A modification was conducted by adding side chains to the PTFE skeleton and to each of these, a terminal sulfonic acid group (-SO_3_H) was also added; this second modification is known as the sulfonation process. Once the chemical modifications are finished, a Nafion^®^ membrane is produced made from a perfluorinated polymer with side chains, resulting in a $SO_{3}^{-}$ group balanced with a $Na^{+}$ ion—so it could be said that it is a sodium salt. The added sulfonated group makes the Nafion^®^ membrane hydrophilic, and can absorb large amounts of water, increasing up to 50% from the dry weight of the membrane. However, under the conditions presented, the membrane still does not acquire its σ; this property is acquired directly via an activation process before its use. This activation process consists of a heat treatment (~80 °C) with concentrated $H_{2}SO_{4}$. This treatment generates Na^+^ that is discarded in the form of sodium sulfate, i.e., for the membrane to acquire PC it must be in its acid form (SO_3_H) [68]. Chemically, the Nafion^®^ membrane has hydrated microdomains, where the $H^{+}$ ions are weakly attracted towards the SO_3_^−^ groups and form hydronium ions (H_3_O^+^) with the water. This process allows the ions to move through the Grotthuss mechanism, where the $H^{+}$ jumps from one microdomain to another [68,69]. 

NF-117 is the model separator used in the different types of MFC [5,40,70]. This choice is due to (i) its high σ, which depends both on the degree of hydration (influenced in turn by temperature and pressure) and on the availability of sulfonic acid groups; (ii) high ionic selectivity, specifically $H^{+}$; (iii) because it is chemically and thermally stable and, (iv) because of the low OR value that translates into generation of a high current density [40,68,71]. Unfortunately, this membrane has certain limitations: (i) high cost. Cost is one of the most important disadvantages since it represents a percentage greater than 50% of the total cost of the system. It is one of the criteria that prevents MFC technology from being scalable [72]; (ii) sensitivity to cations. Sensitivity to cations refers to the chemical nature of the membrane and depending on the composition of the anolyte (heterogenous composition wastewater used as substrate in MFCs), other cations such as $NH_{4}^{+}$, $Na^{+}$, $K^{+}$, $Mg^{2+}$, and $Ca^{2+}$ can be transported through the membrane in addition to $H^{+}$. The transport of cations other than $H^{+}$ depends on the concentration of those cations; Their concentration must be 10^5^ times higher than the $H^{+}$ concentration. Under these conditions, the $H^{+}$ generated as a product of oxidation will accumulate at the anode, acidifying the anolyte. On the other hand, as long as the concentration of other cations does not exceed the indicated concentration (10^5^ times), an accumulation process of these cations may also be generated [70,72,73]. Furthermore, it is important to consider that the ion flow will be established as a function of the concentration gradient. Vélez-Pérez et al. [53] evaluated the co-treatment of municipal wastewater (MWW) and acid mine drainage (AMD) in a DC-MFC using NF-117 as a separator, which showed a pH of 7.29 and 2.50, respectively. The MWW was placed in the anode chamber while the AMD was placed in the cathode chamber. After 120 h of operation, they observed that the pH of the anode chamber decreased slightly to 7.19 while the pH of the cathode chamber increased considerably to 4.08, indicating that the $H^{+}$ concentration decreased due to a possible $H^{+}$ transfer effect from the cathode to the anode; (iii) permeability to oxygen. A significant amount of oxygen can pass through the membrane from the cathode to the anode chamber. The presence of oxygen directly affects the metabolism of anaerobic bacteria at the anode. That results in a reduction in the performance of the MFC as a function of electrical energy production, expressed in terms of the $\eta_{coul}$ [40,70]; (iv) crossover and loss of substrate. The latter reduces the amount of fuel that can be converted into electricity and the substrate available for bacteria. Besides this, it contributes to the formation of biofouling and limits the transfer of $H^{+},$ contributing to acidification of the anode section, which can translate into a loss of bacterial potential and inhibition; (v) restricted use at high temperatures (>80 °C) due to dehydration. Despite this, in MFCs, dehydration at high temperatures is not a limitation; this is because MFCs work at room temperatures, and the dehydration phenomenon in this this type of system is low [39,74]; and (vi) biofouling—a phenomenon that is associated with the adherence of a mixture of substrate with microorganisms and metabolic products of bacteria on the surface of the membrane, generating a barrier between the soluble transferable $H^{+}$ and the membrane [10,70,75]. Taking into consideration some of these disadvantages (mainly the high cost), several researchers have looked for alternatives to replace Nafion^®^ membranes, with the aim of reducing the cost and achieving similar or better characteristics, having NF-117 as a reference membrane for comparison [5,49,56,72,74,76]. 

The cost of NF-117 has undergone drastic changes in the last decade; a considerable price increase has been reported. The cost of NF-117 has reached a price of >1500 USD/m^2^ (Table 2). These changes are due to its excellent properties and market issues based on the demand for this type of separator. NF-117 has become the first choice for FCs and MFCs. This price variation has caused the cost associated with the membrane in configurations of this type of system to increase from 40 to 60% [23,77].

**Table 1:** Nafion^®^ 117 characteristics.

Membrane Properties	Value	References
Proton conductivity (mS/cm)	2.0–9.5	[5,10,72,78]
Thermal stability (°C)	80–90	[37,39,79]
IEC ^a^ (meq/g)	1.23	[37,71]
Water swelling (%)	22–25	[5,37,39]
Thickness (µm)	175–190	[5,39,72,78]
*K*_0_ ^b^ (cm/s)	1.6 × 10^−5^–2.6 × 10^−3^	[70,72,74,75,80]
$D_{O_{2}}$ ^c^ (cm^2^/s)	9.95 × 10^−7^–5.1 × 10^−5^	[5,70,72,74,75]

^a^ Ion exchange capacity; ^b^ Oxygen mass transfer coefficient; ^c^ Oxygen diffusion coefficient.

**Table 2:** Nafion^®^ 117 cost evolution.

Date	Membrane Cost ($/m^2^)	References
2008	700	[81]
2013	1200	[82]
2016	1659	[83]
2016	1733	[56]
2019	1500	[75]
2019	2229	[38]

### 4.2. Anion-Exchange Membranas

Unlike CEMs, AEMs are characterized by allowing “negative ions” to pass through them. They are an important group of membranes and the second most-used group of membranes in MFCs, only after CEMs. The chemical composition of AEMs is characterized by having positive charges (cations) such as $NH_{4}^{+}$, $NHR_{2}^{+}$, $NR_{2}H^{+}$, $NR_{3}^{+}$, $PR_{3}^{+}$, and $SR_{2}^{+}$ (when the membrane has been hydrated) attached to the polymer matrix through which the transfer of negative ions takes place. However, depending on the size of the anion and the lower hydration capacity of the main cation group, a lower anionic conductivity is observed, which translates into low performance of the electrochemical device [10,39].

The PEMs, e.g., Nafion^®^ and PTFE, are generally used as separators in MFCs. These are the binders mostly used by the scientific community. However, both Nafion^®^ and PTFE are not very efficient in the transfer of hydroxide ions, therefore, their efficiency is reduced when used in properly alkaline FCs and favors the intervention of a type of membrane meeting this need. AEM is a polymeric electrolyte that conducts anions such as $OH^{-}$, $Cl^{-}$, $SO_{4}^{2-}$, and its main differentiator is its inhibition of cation transfer (Figure 5b) [84,85,86,87]. The use of an AEM instead of a CEM is based on the existing interference due to the passage of cations, different from the cation of interest ($H^{+}$), through the CEM. This interference reduces the pH in the anode chamber and inhibits microbial activity; consequently, a high pH in the cathode chamber represents a reduction in the cathode potential [87,88]. Fumasep membranes are a group of AEMs; some of them have been evaluated as separators in MFCs [40].

Highly porous, permeable, and inexpensive materials have been tested for implementation as AEMs, e.g., fiberglass and nylon [25,89,90]. Among commercial membranes, there is a commonly used polyvinyl chloride coating that enhances the mechanical stability of the AEM. Each material implemented as an AEM must be analytically supervised to comply with the physicochemical characteristics that other types of membranes, including commercial ones, already meet, e.g., their IEC, their permeability, their power density, and their $\eta_{coul}$ [87,91]. 

Kim et al. [75] reported that the use of AEM in MFCs is reflected in an improvement in power density of 610 mW/m^2^ compared to the typical use of Nafion^®^ at 514 mW/m^2^. 

### 4.3. Bipolar Membranes

A BPM is a membrane made by the union of a CEM with an AEM. These membranes’ objective is to be able to transport $H^{+}$ and $OH^{-}$ simultaneously and contribute to a load balance. There is little information on the use of this type of membranes in MFCs. The main application of a BPM is in electrodialysis processes. In these processes, an electric field dissociates the water molecule into $H^{+}$ and $OH^{-}$ and, immediately, the protons migrate through the CEM while the $OH^{-}$ migrates through the AEM [18].

Heijne et al. [92] used an MFC with a BPM and operated it in continuous mode, achieving ferric iron reduction on a graphite electrode at the cathode (Equation (12)). In addition to the $Fe^{3+}$ remotion, the cell showed a maximum power density of 0.86 W/m^2^ and a $\eta_{coul}$ around 90%. The BPM played a fundamental role in maintaining the pH necessary in the catholyte (<2.5) to keep the $Fe^{3+}$ soluble. The use of a CEM under the conditions used by the authors would generate fouling on the membrane, because the pH would increase and $Fe^{3+}$ would precipitate on the membrane, decreasing or avoiding the proton transfer or charge transfer processes.

(12) $$Fe^{3+}+e^{-}\to Fe^{2+}$$

On the other hand, Kim et al. [93] compared the MFC performance and the $Cr^{6+}$ removal in two DC-MFCs using two different membranes, a PEM, and a BPM. Hexavalent chromium from electroplating wastewater was used as a catholyte. Acetate was used as a substrate for bacteria and an electron source for MFCs. The DC-MFC operated using a BPM showed a power density markedly higher than the power density obtained with the DC-MFC operated using PEMs, at 150.5 mW/m^2^ and 42.7 mW/m^2^, respectively. Furthermore, the removal efficiency of $Cr^{6+}$ to $Cr^{3+}$ (Equation (13)) was significantly higher in the MFC operated with a BPM cell. This is because the BPM managed to maintain the cathodic reaction without a pH decrease in the anodic chamber.

(13) $$Cr^{6+}+3e^{-}\to Cr^{3+}$$

BPMs are membranes that can be used in heavy metal removal processes through bio-electrodeposition, where the pH of the catholyte and the anolyte must remain unchanged.

## 5. Porous Membranes

This type of membrane is considered a low-cost separator compared to the cost of IEMs [10,40] this is a great advantage for porous membranes. Another difference between IEMs and porous membranes is their selectivity to ions. Porous membranes or porous separators are not selectively permeable to ions, whereas IEMs do have a high ion selectivity rate. This characteristic in porous membranes represents a great porous membrane disadvantage [40]. 

The porous membrane’s function is based on their pore size and porosity. Ions ($H^{+}$/$OH^{-}$) and other large molecules are transferred directly through the electrolyte solution that has become embedded in the membrane’s pores. They present a great transfer of ions due to their porosity, which is reflected in a low $R_{int}$ value. However, due to their pore size and porosity, two undesirable phenomena appear: oxygen and fuel crossover. This quickly unleashes another major operating problem over time: biofouling. The only great advantage in terms of performance associated with this type of separator is a low $R_{int}$, which rapidly increases throughout operations along with the appearance of biofouling, causing the performance of the MFC to decrease. In porous membranes, this type of phenomenon occurs to a greater extent than in IEMs, but to a lesser extent than in membrane-less MFCs [40]. Depending on the pore size, at least four types of porous membranes can be applied: UFM, MFM, CMs, and pore filter materials. Glass wool has also been used as a porous membrane. It has a low cost and was used as in SC-MFC for wastewater treatment and bioelectricity generation. Celgard^®^ is another example of a porous membrane [10,39,40].

### 5.1. Ultrafiltration Membranes

This type of membrane has been utilized for the treatment of water and wastewater. UFMs work by separating particulate pollutants based on their different molecular weights. Furthermore, because of its permeability to cations and anions, it is also used in DC-MFCs. However, due to the pore size they have, there are losses of substrate and oxygen, which reflect in low values of *P* and $\eta_{coul}$. Some UFMs evaluated based on their molecular weights were UFM-0.5K, UFM-1K, and UFM-3K; all of them presented a high $R_{int}$ value (especially the UFM-0.5K) and low power density values of 5 mW/m^2^. On the contrary, when the DC-MFC was operated under the same conditions, but with an AEM and a CEM, a better MFC performance was observed (33–38 mW/m^2^) [10,18].

### 5.2. Microfiltration Membrane

The main application of this type of membrane has been as sludge separators for wastewater treatment. It is efficient in processes of filtration and stable for long operation periods. These attractive characteristics have been used to propose and evaluate this type of membrane in MFCs as separators. Its behavior is similar to UFMs; different types of ions and neutral molecules can pass through this membrane due to its porosity. Some examples of cheap MFMs that have functioned in MFCs are nylon mesh, cellulose filters, and polycarbonate filters. Again, MFMs present the same problems reported for UFMs: OD, fuel crossover, and high $R_{int}$ [10,39].

It is necessary to work on solving the three major problems associated with the structure of these types of membranes (UFMs and MFMs) before they can be a viable option for use in MFCs.

### 5.3. Ceramic Membranes

Some materials recently used as membranes or separators in MFCs are CMs [94,95]. These types of membranes have been widely used in different industrial sectors where their use for power generation in high-temperature FCs stands out. CMs hold a customized porosity, permeability, and tolerance to high temperatures; his last feature is irrelevant for application in MFCs. The membrane’s composition gives it a hydrophilic character. The membrane’s composition includes cations such as $Ca^{2+},\;Mg^{2+},\;K^{+},Na^{+},\;H^{+},$ and $Al^{3+}$, where the first four cations are referred to as the base cations, whereas the last two are referred to as acidic cations. Thus, the total amount of acidic cations present at the CM plays an important role in the MFC in proton transfer [10].

One of the first works reporting the use of CMs was published by Park and Zeikus [95]. They used a ceramic separator on an SC-MFC utilizing different mediators and three-electrode settings at pH 7. The reported maximum current density and power density production were 1750 mA/m^2^ and 788 mW/m^2^, respectively, using sewage sludge as the biocatalyst. On the other hand, Behera and Ghangrekar [96] also demonstrated the potential of CMs as separators in MFCs, generating a volumetric power output of 16.8 W/m^3^. In the same year, Behera et al. [94,97] used commercial earthen pots to test their efficiency, both in pollutant removal and in the production of electricity at different pHs using WW from rice mills as substrate. The chemical oxygen demand removal efficiency $\left(\eta_{COD}\right)$ was 96.5% whereas the maximum sustainable volumetric power was 2.3 W/m^3^ with 100 Ω external resistance. Winfield et al. [98] investigated the properties of ceramic and terracotta materials, focusing on the thickness of the wall, the porosity of the material, and the hydration of the cathode. They showed that the cylindrical mud MFCs produced significantly higher current and power. In the same year, another study was conducted using eco-friendly materials [99]. From this study, two ion exchange materials were utilized: compostable starch-based bags (BioBag) and ceramic with a commercially available cation exchange membrane. The starch bags proved to be an effective material for the microbial environment despite their limited life span (8 months). This finding highlights porous separators as candidates for MFC practical application in terms of cost and operational stability. Recently, Pasternak et al. [75] compared different low-cost CMs used in MFCs against the performance of a commercial PEM. The findings were quite good. The highest volumetric power of 6.93 and 6.85 mW/m^3^ was obtained by using pyrophyllite and earthenware, whereas mullite and alumina produced a volumetric power of 4.98 and 2.60 mW/m^3^, respectively. These results prove the potential of CMs to be used as alternative separators instead of expensive commercial PEMs.

The $R_{int}$ increase in the long-term performance of MFCs is one of the major concerns of this type of membranes. Once again, as a product of the possible biofouling generated [10].

According to Winfield et al. [100], CMs used as separators in MFCs are a viable low-cost, sustainable, and widely available alternative. Another advantage of ceramic spacers is that they provide the chassis of the MFCs and produce catholyte. They proved that a less dense material generates a higher *I* output. Another study carried out by Merino-Jimenez et al. [101,102] reports advances using ceramic cylinders with different porosities.

### 5.4. Coarse-Pore Filters

Any permeable material that does not impede charge transfer and has insulating characteristics to prevent short circuiting between electrodes can be used as a separator in MFCs. Taking this into consideration, some of the materials that have been used as coarse-pore filters in the architecture of devices are the following: porous fabrics, glass fiber, cellulose filters, agar–agar membranes, nylon mesh, and non-woven paper fabric filters [24,25,56,103]. These materials are cheaper and show high potential for practical application. However, some of these materials may allow greater oxygen and substrate exchange, affecting the device’s performance due to biofouling generation [104].

One of the materials reported for potential replacement of polymeric membranes in MFCs is fabric. This material was used for the first time in the work of Fan et al. [105]. The authors report having used J-cloth in an SC-MFC, reducing the $R_{int}$ and resulting in a volumetric power of 627 W/m^3^ operating in batch mode and 1010 W/m^3^ in continuous flow. Another type of fabric reported as a potential effective separator, due to proton transfer, is canvas. Zhuang et al. [106] reported the use of canvas in cathode assembly, using conductive paint based on nickel or graphite and a catalyst (MnO_2_). The SC-MFC they designed operated with brewery wastewater for 13 and 18 days in batch mode, generating power densities of 86.03 and 24.67 mW/m^2^ (normalized to the cathode surface) or of 9.87 and 2.83 W/m^3^ (normalized to the volume of the liquid), respectively. Furthermore, with an external resistance of 100 Ω, a $\eta_{COD}$ and a $\eta_{coul}$ of 95 and 30.2%, respectively, were reached.

Zhang et al. [104] experimented with inert and non-biodegradable materials to reduce the OR and increase the *P*, favoring the transport of protons to the cathode. In their work, they compared the performance of a CEM with different configurations of fiberglass and J-cloth-based separators. The latter degraded over time and showed less favorable results than fiberglass. The highest volumetric powers were obtained in the devices that used fiberglass separators (46 ± 4 W/m^3^) and J-cloth (46 ± 1 W/m^3^). The implementation of spacers allows a reduction in the distance between the electrodes and therefore, improves the volumetric power density, as long as it is possible to reduce the fuel and oxygen crossover that triggers the biofouling formation, and, finally, a deterioration in MFC performance.

Choi et al. [103] evaluated a non-woven paper fabric filter (*NWF*) as a separator in an MFC. The MFC performance was compared with an MFC using an NF-117 as a separator. The MFC using the NWF showed a volumetric power of 1027 mW/m^3^, whereas the MFC equipped with NF-117 reached a volumetric power of 609 mW/m^3^. Moreover, the MFC with an NWF showed stable cell performance (550 mV) over 300 days. On the other hand, the MFC with an NF-117 showed biofilm formation and chemical precipitation on the membrane surface. In consequence, the voltage decreased from 551 to 415 mV. This phenomenon could be associated to the acetate mass transfer coefficient. The acetate mass transfer coefficient for the NWF was slightly lower than that of PEM, at 1.6 × 10^−4^ and 2.2 × 10^−4^ cm/s, respectively. Finally, another point in favor for NWF was the cost. The cost per square meter was less than 4 USD/m^2^, whereas the cost reported for NF-117 in that study was 1400 USD/m^2^.

## 6. Membrane-Less Microbial Fuel Cells

In *PE*MFCs, the separation of H_2_ and O_2_ gases is essential. This important task is carried out by a membrane that must be able to conduct the protons produced by the oxidation of H_2_ (Equation (1)) towards the cathode, for the corresponding simultaneous reduction of O_2_ (Equation (2)). A CEM, also referred to as a PEM for its assigned function (proton transfer), is the membrane of choice. However, in the case of MFCs, the membrane could be an unnecessary component of the system configuration. MFCs without a membrane were one of the first configurations used in the principle of MFCs [16,49]. This modification to the system is based on the property of water transfer of $H^{+}$ from the anode chamber to the cathode chamber directly through the system [18,107,108].

As previously analyzed, the use of nonporous membranes or porous membranes generates a series of electrochemical, microbiological, and physicochemical problems within MFCs. The main factor affected by the presence of a membrane or separator is the $R_{int}$. By itself, the membrane has an *R* value. When placed inside an MFC, this value is added to the other resistances of the system, increasing $R_{int}$ [49,109]. However, the value of *R* is not constant within the system. Its value changes negatively for the system throughout the operation time because of the biofouling formation that indirectly increases the thickness of the membrane; therefore, the value of $R_{int}$ increases even more. Besides, biofouling decreases the σ, generating another phenomenon—pH splitting [66,75]. Thus, the use of the membrane and its associated effects causes MFC performance to decrease. Another important parameter that is not related to the MFC’s performance is related to its cost. Depending on the type of membrane chosen for use in an MFC, the construction and operation costs increases considerably by up to 60% if NF-117 is used [23,40]. Due to the negative effects associated with the presence of membranes within the configuration of MFCs and the ability of water to transfer protons, the option of not using membranes and operating the MFCs without the use of them has been considered [1,15]. Furthermore, the elimination of membranes or separators in the design of MFCs responds to the need to reduce the cost of the devices and simplify their design, both of which are critical factors for the scaling and application of these devices in real scenarios [1,110,111,112,113,114]. 

One of the first experiments conducted on membrane-less SC-MFCs was reported by Liu & Logan [15]. The authors experimented with one device using NF-117 and another without a spacer between the carbon electrodes. Domestic wastewater (WW) and glucose were used as substrate. In their results, they report that by removing the CEM, the power density increased to 494 ± 21 mW/m^2^ (12.5 ± 0.5 mW/L). On the other hand, the $\eta_{coul}$ was 40–55% using CEM and 9–12% without it. This remarkable difference in $\eta_{coul}$ values is attributed to a substantial diffusion of oxygen towards the anode due to the lack of a barrier. In later works, Liu et al. [115] experimented with scaling the membrane-less SC-MFC up to 520 mL operating in batch mode. With this volume, they obtained 16 W/m^3^. Increasing the ionic strength of the device from 100 to 300 mM using NaCl raised the volumetric power by 25% to 20 W/m^3^. When the device was operated in a continuous flow, a volumetric power of 22 W/m^3^ was reached with a hydraulic retention time of 11.3 hours. Their results showed that the output power can be maintained in reactors with the indicated volume and that the separation of the electrodes is a determining factor for power generation [115]. 

Santoro et al. [116] reported the first membrane-less SC-MFC operated in batch mode with human urine. For its configuration, a modified glass bottle with a side hole was utilized where the cathode was connected; this component was used with and without Pt coverage.

When membrane-less MFCs are connected in series, the arrangement is inefficient, because it is not possible to equalize the sum of the Δ*V* produced by the individual cells. Therefore, these devices require design improvements [93]. However, at the start of the operation of a membrane-less MFC, the main effect observed is on the $R_{int}$; its value decreases considerably and in turn is reflected in a notable increase in the *P_MFC_* [117]. Additionally, a lower investment cost in the construction of membrane-less MFCs is attractive [10,39]. Despite the apparent advantages of operating MFCs without membranes, in relatively short operating times, the price of not using a membrane is reflected in a considerably lower value of $\eta_{coul}$ compared to a cell operated with a membrane. Without the membrane, the proton transfer rate from the anode to the cathode is high and two undesirable phenomena appear due to the lack of a barrier between the two half-reactions: fuel crossover and OD. The highest fuel and oxygen crossover values have been observed in this type of configuration. OD and fuel crossover are mainly responsible for the considerable decrease in MFC performance in terms of $\eta_{coul}$ [107,108].

As previously discussed, the presence of oxygen at the anode competes for the electrons generated by oxidation. Besides, the crossover fuel towards the cathode decreases the anodic fuel load available to be anaerobically oxidized by electrochemically active bacteria and harvests the electrons through the external circuit of the system [1,2,15,18,25,118]. The fuel crossover generates a biofilm on the cathode surface, and the interaction of oxygen with the cathode surface decreases, i.e., the biofilm prevents oxygen from acting as an electron acceptor at the cathode [119]. Also, in membrane-less MFCs values of $\eta_{COD}$ > 90% have been reported—a significantly high $\eta_{COD}$ value. However, this $\eta_{COD}$ is attributed to a mainly aerobic oxidation process. Organic matter is oxidized under aerobic conditions at the cathode, therefore, the oxidation rate and $\eta_{COD}$ are higher [40]. In the work of Ghangrekar and Shinde [110], the performance of a device without mediators or membranes using synthetic and raw *WW*, an $\eta_{COD}$ ca. 90% was achieved. The high value of $\eta_{COD}$ does not justify the operation of MFCs. If this were the case, we would be talking about aerobic oxidation systems that do not need to be operated or be constructed as MFCs, but as simple aerobic oxidation systems where the process is carried out within the same system. In summary, the presence of a membrane greatly reduces the phenomena of fuel and oxygen crossover, improving the $\eta_{coul}$ [15]. The use of a membrane in MFCs is not essential; nevertheless, the researchers concluded that a separator or membrane is necessary to ensure an efficient and sustainable MFC operation [18,34].

## 7. Salt Bridge

In the beginning of MFC technology development, a salt bridge was utilized instead of a membrane separator [49,120]. The salt bridge is a form of separator used specifically in DC-MFCs. It leads the ions through a tube filled with electrolytes. Typically, inert electrolytes are used, such as saturated solutions of KCl and phosphate buffer solution, while agar, an organic polymer, is added to counteract the exchange of fluids [16,121]. The elaboration of a salt bridge as a separator is cheap, even more so than porous membranes. However, the MFC performance results obtained with the salt bridge have not been very encouraging. The power output is usually one order of magnitude lower when using devices with a salt bridge compared to using NF-117 as a separator. This difference in power is attributed to the high value of $R_{int}$ observed for the MFC using a salt bridge as a separator, ca. 20,000 Ω, while the $R_{int}$ observed for the MFC operated with NF-117 was significantly lower, ca. 1300 Ω. Furthermore, in both systems, using NF-117 and the salt bridge, the OD phenomenon was observed from the cathode to the anode [16]. Besides, Kargi and Eker [17] evaluated a DC-MFC to generate bioelectricity and wastewater treatment using a low-cost separator: a salt–agar slab. However, copper and gold-covered copper wires were used as anodes and cathodes, respectively. The results showed a low power density, ca. 3 mW/m^2^.

The thickness of the materials used as spacers is one of the determining factors for the OR value. The separator OR high value is reflected in a high value of $R_{int}$ which, in turn, affects the $I_{MFC}$ and $P_{MFC}$ (Equations (5)–(7)). The *R* value is directly proportional to the thickness and inversely proportional to the area of the spacer, as shown in Equation (14):

(14) $$R=\rho \frac{L}{A}$$

where *R* is the membrane or separator resistance (Ω), ρ is the material resistivity (Ω·m), *L* is the separator thickness (m), and *A* is the surface area of the electrode (m^2^).

Based on the salt bridge composition, Hernández-Flores et al. [38,52,56] synthesized and evaluated membranes based on different concentrations of agar–agar and KCl. The reported cost of the proposed membranes based on their composition ranged from 9 to 47 USD/m^2^—two orders of magnitude lower than the price reported for the commercial NF-117 membrane in the last decade (Table 2). In general, these membranes presented with small thicknesses (274–1100 µm) and high PC values (1.13–7.10 mS/cm), since they were subjected to a dehydration process to reduce *R* (Equation (14)). However, the membranes containing KCl presented with an OD problem due to the dissolution of KCl in an aqueous medium. Unlike a conventional salt bridge, these membranes were used in SC-MFCs, and, comparing against NF-117 under the same operating conditions, encouraging results were found in terms of a power–cost analysis that was carried out [56]. On the other hand, the MFC performance using a 2% *w*/*v* agar–agar membrane and leachates from a sanitary landfill as substrate, reached a volumetric power of 20,000 mW/m^3^, whereas the commercial NF-117 membrane operating under the same conditions reached a volumetric power of 6800 mW/m^3^ [52].

Salt bridge use is limited to DC-MFCs only. Also, their use represents high values of $R_{int}$ and low values of *P*. For that reason, and despite their low cost; they have not continued to be utilized as separators. On the other hand, agar–agar membranes are inexpensive and have shown low $R_{int}$ values and encouraging volumetric power values in SC-MFCs.

## 8. Conclusions and Outlook

Studies on MFCs as new green eco-friendly technology have awoken huge interest. Although their performances are low compared to the *Ps* generated by PEMFCs, the scientific community continues to look for improving the MFC performance. The interest in this technology is based on potential WW treatment under anaerobic conditions and the generation of bioelectricity, i.e., the conversion of organic pollutants into energy and the generation of effluent with a lower content of organic matter. Nevertheless, several factors directly impact MFC performance. The membrane or separator used in these devices represents a key part of proper operation and good performance. So far, Nafion^®^ membranes (perfluorinated membranes), a type of PEM, have been shown to have the best characteristics in terms of performance ($\eta_{coul}$ and $P_{MFC}$) for MFCs. However, an important issue that membranes present during their operation is their high cost, which limits their application. Despite the high cost, the NF-117 membrane is the model membrane in MFCs. The main properties of the Nafion^®^ membrane are relatively high σ, chemical stability, and excellent mechanical properties. The search to synthesize membranes or separators of low cost and with attractive performances as alternatives to NF-117 is still in progress. Some considerations discussed previously must be taken into account to propose new alternative membranes or separators. The development of new alternative membranes to apply in MFCs is a difficult task. However, finding a new membrane able to reach competitive MFC performance at a low cost is necessary for scaling up. A membrane must perform a lot of characteristics to be considered as an ideal membrane.

A membrane could be considered ideal for use in MFCs if it has the following properties: a high σ, low water and substrate loss, low thickness, impermeability to oxygen and cations such as $NH_{4}^{+}$, $Na^{+}$, $K^{+}$, $Mg^{2+}$, and $Ca^{2+}$, good mechanical properties, chemical stability, low $R_{int}$ values, impermeability to gases such as $H_{2}$, $N_{2}$, and, definitely, a low price. Meeting all these properties simultaneously is difficult. However, this search focuses on finding membranes that have the greatest number of positive attributes and, above all, whose use reflects a good MFC performance in terms of low average $R_{int}$, long stability or durability over the operation time under different operating conditions, biofouling resistance, being nonbiodegradable, low cost, and high $\eta_{coul}$. Unfortunately, there is no ideal membrane. The IEMs, especially the PEMs, are the membranes that depict some of the most important properties, and this allows the membranes to be applied directly in MFCs. The proposed porous membranes and other separators are not the best choices as ion-exchange separators to be used in MFCs.

The presence of a membrane as a separator in MFCs is not essential. However, it significantly improves MFC performance, although its use has a direct impact on initial investment and maintenance costs. Again, a trade-off solution should be accomplished. Although the use of membranes increases the cost, it improves performance. Finally, despite all the advantages of MFCs (wastewater treatment, bioenergy generation as bioelectricity, $CH_{4}$ or $H_{2}$, and heavy metals removal), the low *Ps* and the cost of the membranes still represent a challenge for a suitable scaling up of this technology. The presence of membranes or separators as a part of MFC configurations contributes to the operation of the MFCs for prolonged periods and favors the electrochemical performance. Thus, although the use of a membrane shows several drawbacks as part of the MFC configuration, its use is highly recommended. There are many membranes proposed for use in MFCs. However, the characteristics and performances observed lead us to suggest that the best strategy is to develop membranes focused on the synthesis of IEMs using low-cost materials (organic polymers) with high species selectivity and low *R,* but high biofouling resistance and good chemical stability.

## Data Availability

The data presented in this study is based on a literature review of published materials and are available on request from the corresponding author.

## Abbreviations

This is a list with the acronyms used through the manuscript. It will help the reader to easily understand the text.

*A*	Surface area of the electrode
*ACS*	Actual charge due to the substrate
AEM	Anion exchange membrane
AMD	Acid mine drainage
*b_COD_*	Number of electrons exchanged per mole of oxygen
BPM	Bipolar membranes
CEM	Cation exchange membrane
*CI*	Current interruption
CM	Ceramic membrane
*COD*	Chemical oxygen demand
*COD_f_*	Final chemical oxygen demand
*COD_i_*	Initial chemical oxygen demand
DC-MFC	Dual-chamber microbial fuel cell
*D_O2_*	Oxygen diffusion coefficient
*E_MFC_*	Voltage generated by the microbial fuel cell
*F*	Faraday’s constant
*FC*s	Fuel cells
*I*	Current
IEC	Ion-exchange capacity
IEM	Ion-exchange membrane
*I_MFC_*	Electrical current of the microbial fuel cell
*K_O_*	Oxygen mass transfer coefficient
*L*	Membrane thickness
*M_COD_*	Molecular weight of oxygen
MFC	Microbial fuel cells
*MFM*	Microfiltration filtration membrane
MWW	Municipal wastewater
NF-117	Nafion^®^ 117
OD	Oxygen diffusion
OR	Ohmic resistance
*ORR*	Oxygen reduction reaction
*P*	Power density
PC	Proton conductivity
PEM	Proton-exchange membrane
*PE*MFC	Proton-exchange membrane fuel cell
PFSA	Perfluorinated sulfonic acid
*P_MFC_*	Power generated by the microbial fuel cell
PTFE	Polytetrafluoroethylene
*R*	Membrane resistance
*R_int_*	Total internal resistance of the microbial fuel cell
SC-MFC	Single chamber microbial fuel cell
*SPEEK*	Sulphonated polyether ether ketone membrane
*TCS*	Theoretical charge due to the substrate
*UFM*	Ultrafiltration membrane
*V*	Volume of liquid in the anode compartment
*WW*	Wastewater
*η_coul_*	Coulombic efficiency
*η_COD_*	Chemical oxygen demand removal efficiency
Δ*V*	Voltage difference
*ρ*	Resistivity
